# Potential anti-dengue medicinal plants: a review

**DOI:** 10.1007/s11418-013-0767-y

**Published:** 2013-04-17

**Authors:** Siti Latifah Abd Kadir, Harisun Yaakob, Razauden Mohamed Zulkifli

**Affiliations:** 1 Department of Bioprocess Engineering, Faculty of Chemical Engineering, Universiti Teknologi Malaysia, 81310 Skudai, Johor Malaysia; 2 Institute of Bioproduct Development, Universiti Teknologi Malaysia, 81310 Skudai, Johor Malaysia; 3 Department of Biological Sciences, Faculty of Biosciences and Bioengineering, Universiti Teknologi Malaysia, 81310 Skudai, Johor Malaysia

**Keywords:** Dengue fever, Anti-dengue, Medicinal plants, Phytochemical

## Abstract

Dengue fever causes mortality and morbidity around the world, specifically in the Tropics and subtropic regions, which has been of major concern to governments and the World Health Organization (WHO). As a consequence, the search for new anti-dengue agents from medicinal plants has assumed more urgency than in the past. Medicinal plants have been used widely to treat a variety of vector ailments such as malaria. The demand for plant-based medicines is growing as they are generally considered to be safer, non-toxic and less harmful than synthetic drugs. This article reviews potential anti-dengue activities from plants distributed around the world. Sixty-nine studies from 1997 to 2012 describe 31 different species from 24 families that are known for their anti-dengue activities. About ten phytochemicals have been isolated from 11 species, among which are compounds with the potential for development of dengue treatment. Crude extracts and essential oils obtained from 31 species showed a broad activity against *Flavivirus*. Current studies show that natural products represent a rich potential source of new anti-dengue compounds. Further ethnobotanical surveys and laboratory investigations are needed established the potential of identified species in contributing to dengue control.

## Introduction

### Etiology of dengue fever

Dengue fever is caused by the arthropode-borne flavivirus named dengue virus (DENV), transmitted by the *Aedes aegypti* mosquito [1]. To date, four antigenically related but distinct virus serotypes (DENV-1, 2, 3 and 4) have been identified as belonging to the genus *Flavivirus* in the Flaviviridae family [2–4]. Infection with one DENV serotype produces only specific antibody against that serotype. When antibody from the first infection is neutralized, secondary infections by other serotypes can cause more serious infection [5]. Although DENV-2 is known to be more lethal than other serotypes [6], some studies have revealed that primary infection with DENV-1 or DENV-3 always results in more dangerous disease than infection with DENV-2 or DENV-4 [3, 7]. In recent years, the current dengue epidemic has become a focus of international public health awareness. Unlike malaria, which is more prevalent in remote areas, cases of dengue are distributed mostly in urban and sub-urban areas [8, 9]. This has made the epidemic more lethal as an outbreak is difficult to control due to highly populated areas in cities.

Types of DENV infection include mild fever known as dengue fever (DF), which constitutes about 95 % of cases, and a more serious type known as dengue hemorrhagic fever and/or dengue shock syndrome (DHF/DSS, 5 % of cases) [10, 11]. Recovery from first type of infection provides lifelong immunity; however, it affords only half protection from subsequent viral infection that ultimately results in the risk of DHF. Most dengue infections are characterized by non-specific symptoms including frontal headache, retro-orbital pain, body aches, nausea and vomiting, joint pains, weakness and rash [12, 13].

### Epidemiology of dengue fever

**Fig. 1:**
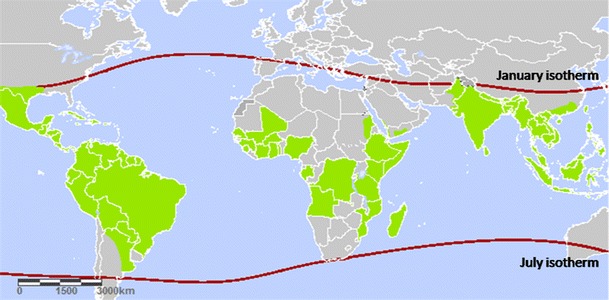
*Green* Countries or areas at risk of dengue, 2012. The *contour lines* of the January and July isotherms indicate the potential geographical limits of the northern and southern hemispheres for year-round survival of *Aedes aegypti*, the principal mosquito vector of dengue viruses. This copyrighted map is reproduced with acknowledgment to the World Health Organization (WHO)

International travel, increasing human population [14, 15] and urbanisation create suitable conditions for the mosquito vector *Ae. aegyti*, and thus spread the virus to new areas, causing major epidemics [13, 16, 17]. Dengue epidemics are endemic in over 100 countries in Africa, America, Eastern Mediterranean, Southeast Asia and Western Pacific, with Southeast Asia and the Western Pacific being the regions most affected (Fig. 1) [13, 18–20]. The first case of DHF was discovered in the 1950s in Thailand and the Philippines [4], where the first two DENV serotypes were identified, followed by the third and fourth serotypes in 1954 [14]. Since then, DHF has recorded major cases resulting in hospitalization and death among children in regions stretching from Asia to Africa and the Pacific [4]. Approximately 2.5 billion people, or half the world’s population [14], are now at risk of Dengue, and 50 million infections globally occur annually [4]. Over 100 million cases of DF and at least 500,000 cases of DHF [21] and approximately 18,000 deaths may occur each year [22]. Despite its lethal consequences, the staggering numbers of those affected are increased by the fact that, at present, there is no specific antiviral treatment or vaccine for DF [3]. Early diagnosis and strict hospitalization often save the life of patients with DHF [3, 4, 10]. Efforts to combat the vector have been undertaken by regulatory bodies in an attempt to tackle this problem by awareness campaigns and vector control [16]. Others strategies include the use of plants with bioactive substances that have toxic properties to the vector or insecticidal properties [20]. Clearly, development of antiviral drugs and vaccines is needed in order to support these programs. Moreover, a safe, low-cost, and effective vaccine to control DENV woudl be needed, especially in the most affected countries, which are poor [2, 16]. Therefore, the search of highly selective but non-toxic antiviral compounds is urgently needed in view of the spread of dengue disease throughout the world [23].

## Global distribution of dengue fever

Guangdong province in China has become a major area with reported cases of dengue [24]. From 2000 to 2005, a total of 2,496 cases of dengue were recorded. The epidemic peaked in 2002. In Northern Thailand there were 13,915, 11,092, 6,147, 6,992 and 6,914 DF cases reported during the period 2002–2006 [25]. Outbreaks of DF and DHF have been reported in India over the past four decades [26]. From 2001 to 2002, Delhi recorded a decline in cases of DF/DHF, with a total of 1,380 cases, but deaths decreasing from 53 cases (2001) to 35 cases (2002). However, outbreaks of DF cases rose sharply in 2003, with a total of 12,754 cases and 215 deaths.

### Dengue fever in Malaysia

**Fig. 2:**
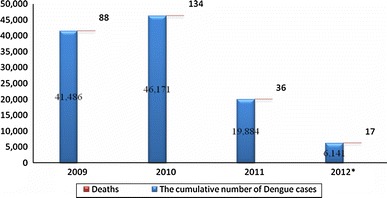
Reported dengue cases in the years 2009–2012 in Malaysia [32–36]. *Data up to 24 March 2012

In Malaysia, with a population of 27.7 million and a population density of 84 per km^2^ [27], outbreaks of dengue cases are endemic, with increasing cases of dengue over the past two decades. The first case was documented in 1902 [16, 28, 29]. During the period 1973–1982, 12,077 dengue cases were reported, with a fatality rate of 3.38 %. The number of cases rose in following decade of 1983–1992 with 26,361 cases; however, the fatality rate was down to 0.55 % [28]. In 2004 and 2005, dengue was reported with 13,558 and 15,862 incidence rate, respectively, per 100,000 population. With an increase of 16.99 % of cases, a total of 107 deaths were recorded in 2005 compared to 102 cases in 2004 [29]. According to Health Facts 2006 (Ministry of Health Malaysia), the incidence rates of DF and DHF were 64.37 and 4.10 per 100,000 population, respectively, with mortality rate of 0.01 (DF) and 0.25 (DHF) [30]. In a press statement, the Director General of Health Malaysia, reported a total of 545 cases and four deaths in 5 weeks in 2012 as the highest increase of dengue cases and deaths, with an increase of 57 cases (12 %) compared to 488 cases with two deaths the previous week [31]. In the period 2009–2011, the number of dengue cases decreased to 21,602 cases with the peak appearing in 2010 (Fig. 2) [32–36].

Since early human civilization, plants have been a source of traditional medicine, and demands for herbal and natural product have recently increased. About 70–95 % people worldwide now rely on traditional herbs as the primary treatment for various diseases [37]. It is estimated that about 25 % of modern drugs, including antiviral agent, originate from natural products [38] with over 60 % of anti-cancer compounds and 75 % of infectious disease drugs being derived from natural ingredients, which are more acceptable, less toxic and less expensive than synthetic drugs [39, 40]. Several studies have reported potential antiviral agents from plants in the form of crude extracts, essential oils or purified compounds [41, 42]. Recent studies have reported the potential of some flavonoid compounds as antivirals against DENV-2 [40, 43].

### Pathophysiology of dengue fever

Dengue infection is caused by bites of the female *Ae. aegypti* mosquito carrying *Flavivirus*. After a person is bitten, the virus incubation period varies between 3 and 14 days [3, 18], after which the person may experience early symptoms such as fever, headache, rash, nausea, and joint and musculoskeletal pain [3, 13]. This classic DF records temperatures between 39 and 40 °C and usually lasts 5–7 days [6]. During this period, the virus may get into the peripheral bloodstream and, if left untreated, can damage blood vessels and lymph nodes resulting in DHF with symptoms such as bleeding from the nose, gums or under the skin [18]. DHF patients also have difficulty in breathing and severe development can lead to DSS. DSS can result in death if proper treatment is not provided.

**Fig. 3:**
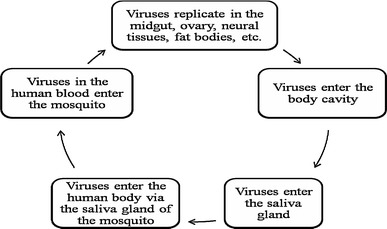
Dengue virus transmission cycle

**Fig. 4:**
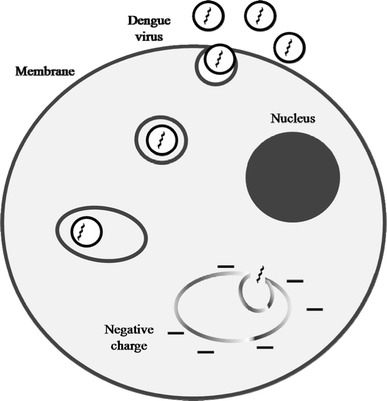
Dengue virus infection cycle in cells

*Aedes* mosquitoes are small and black with white markings on the body and legs. Female mosquitoes need blood from biting humans or animals to produce live eggs. It takes 2–3 days for egg development. The principal vector of dengue (*Ae. aegypti*) has adapted well to the urban environment [14, 17] and always breeds in stagnant containers. Eggs need moist conditions, and mature in 24–72 h [44]. Mosquito bites are the only route of DENV spread. The transmission of DENV is often from human to human through domestic mosquitoes [6]. An outbreak starts after a mosquito sucks the blood of a patient with DF/DHF (Fig. 3) [44]. After being transmitted to a new human host by infected mosquitoes, the virus replicates in the lymph nodes and spreads through the lymph and blood to other tissues [6]. To identify a potential antiviral treatment for DENV, it is necessary to understand the life cycle of the virus. The dengue virion is a small particle with a lipoprotein envelope and an icosahedral nucleocapsid containing a positive single-stranded RNA genome [6, 12, 23]. Virus infection of the cell begins with binding to the host cell surface. It enters the cell by receptor-mediated endocytosis [15], with the cell membrane forming a sac-like structure known as an endosome. In the endosome, the virus penetrates deep into the cell until the endosome membrane acquires a negative charge, which allows it to fuse with the endosomal membrane to open a port for release of genetic material. At this point, the virus in the cell fluid starts to reproduce. Changes in the acidity of the secretory pathway during this viral journey travel play an important role in its maturation (Fig. 4).

### Possible mechanisms and pathways in the treatment of dengue

There are currently no specific treatments for dengue fever [22]. Only standard treatment for management of fever is given, i.e., nursing care, fluid balance, electrolytes and blood clotting parameters [18]. Patients with dengue fever will be treated symptomatically, for example, sponging, acetaminophen [9], bed rest and oral rehydration therapy, and if signs of dehydration or bleeding occur the patients are usually hospitalized [6]. Aspirin should be avoided because it may cause bleeding [9]. Platelet count and Hematocrit should be measured daily from the suspected day of illness until 1–2 days after defervescence [9]. Current prevention of dengue by potential dengue vaccine and vector control is highly cost effective [22, 45]. In addition, mosquito control programs are the most important preventive method [6]. However, these are difficult to implement and maintain [39]. Development of a vaccine for dengue is difficult since there are four closely related, but antigenically distinct, serotypes of the virus that can cause disease [6, 46]. Infection by one serotype does not ensure protection of the patient from infection by the other three serotypes [15]. Therefore, if vaccine were produced for only one or two serotypes, the other serotypes would increase the risk of more serious illness [47]. Ribavirin has shown significant in vivo activity against RNA viruses; however, it exhibited only very weak activity against *Flaviviruses* [21]. A possible strategy in the treatment of dengue is to use chimeric tetravalent vaccines that show high neutralizing antibody against all dengue serotypes [9, 15]. Studies on the development of tetravalent vaccines are ongoing in Thailand and these should be available in the near future [6]. In addition, recombinant vaccines against capsid, premembrane and envelope genes of DENV-1, -2 and -3 inserted into a copy of a DNA infectious clone of DENV-2 are being developed and are currently undergoing clinical trials [48].

### Plants traditionally used to treat dengue

According to a World Health Organization (WHO) fact sheet dated December 2008, 80 % of the population in some Asian and African countries depends on traditional medicine as their primary health care due to economic and geographical constraints [49]. Natural products have become the main source of test material in the development of antiviral drugs based on traditional medical practices [50]. Traditional medicines are based on knowledge, experience and practices based on indigenous cultural beliefs and knowledge, and are used to maintain health, prevent, treat and diagnose physical or mental illness [49]. Traditional medicinal plants have been reported to have antiviral activity [49, 51, 52] and some have been used to treat viral infections in animals and humans.

To date, 31 different species have been found to have the potential to treat dengue; some of these have not yet been investigated scientifically (as indicated in Table 1). In the Philippines, *Euphorbia hirta*, known locally as “tawa–tawa”, is used in folk medicine to cure dengue fever by people in rural areas [53]. Practitioners of traditional medicines believe that decoction of tawa–tawa leaves can reverse viral infection and prevent the fever from moving into critical stages, although there are no scientific studies proving its effectiveness [54]. Sometimes, tawa–tawa is prepared together with papaya leaves since papaya leaf extract has a function as an antibiotic to cure fever. While papaya leaf extract kills the bacterial infection that caused the fever, tawa–tawa extract prevents bleeding. In addition, unpublished research has found that *Psidium guava* leaves are a good way to increase platelets, thus helping to avoid bleeding [55]. A water decoction of guava leaves contains quercetin, which acts to inhibit the formation of enzyme mRNA in the virus [56].

**Table 1:** Plants with reported anti-dengue activity, according to family

Family	Species	Local/common name	Part(s) used	Compound isolated	References
Acanthaceae	*Andrographis paniculata*	Hempedu Bumi (Malaysia)	Leaves		[7]
Amaranthaceae	*Alternanthera philoxeroides*	Alligator weed	Whole plants		[57]
Caricaceae	*Carica papaya*	Papaya	Leaves		[9]
Chordariaceae	*Cladosiphon okamuranus*	Brown seaweed	Whole plants	Fucoidan (**3**)	[59]
Cucurbitacea	*Momordica charanthia*	Bitter Melon, Peria (Malaysia)	Fruit		[7]
Elaeagnaceae	*Hippophae rhamnoides*	Sea Buckthorn	Leaves		[11]
Euphorbiaceae	*Cladogynos orientalis*	Chettaphangkhee (Thailand)	Whole plants		[2]
	*Euphorbia hirta* ^a^	Gatas–gatas	Leaves		[53, 54]
Fabaceae	*Leucaena leucocephala*	White Leadtree, Petai Belalang (Malaysia)	Seeds	Galactomanan (**7**)	[12, 62]
	*Mimosa scabrella*	–	Seeds	Galactomanan (**7**)	[12]
	*Tephrosia madrensis*	–	Leaves and flowers	Glabranine (**8**), 7-*O*-methylglabranine (**9**)	[10]
	*Tephrosia crassifolia*	–	Leaves and flowers		[10]
	*Tephrosia viridiflora*	–	Leaves and flowers		[10]
Fagaceae	*Quercus lusitanica*	Gall Oak	Seeds		[39]
Flagellariaceae	*Flagellaria indica*	Whip Vine	Whole plants		[2]
Halymeniaceae	*Cryptonemia crenulata*	Red seaweed	Whole plants	Galactan (**4**)	[60]
Labiatae	*Ocimum sanctum*	Holy Basil, Tulsi (India)	Leaves		[7, 64]
Meliaceae	*Azidarachta indica*	Neem	Leaves		[8]
Myrtaceae	*Psidium guajava* ^a^	Guava, Jambu Batu (Malaysia)	Leaves		[55, 56, 66]
Piperaceae	*Piper retrofractum*	Dīplī (Thailand), Long Pepper	Whole plants		[2, 65]
Phyllophoraceae	*Gymnogongrus torulosus*	Red seaweed	Whole plants	Galactan (**4**)	[61]
	*Gymnogongrus griffithsiae*	Red seaweed	Whole plants	Kappa carrageenan (**5**)	[60]
Poaceae	*Cymbopogon citratus*	Lemon Grass	Whole plants		[7]
Rhizophoraceae	*Rhizophora apiculata*	Bakau (Malaysia)	Whole plants		[2]
Rubiaceae	*Uncaria tomentosa*	Cat’s Claw	Stem barks		[67, 68]
Saururaceae	*Houttuynia cordata*	Pak Kan Thong (Thailand), Chameleon Plant	Whole plants, aerial stem and leaves	Hyperoside (**6**)	[2, 5]
Solieriaceae	*Meristiella gelidium*	–	Whole plants	Kappa carrageenan (**5**)	[63]
Verbenaceae	*Lippia alba*	Pronto Alivio (Colombia), Bushy Matgrass	Whole plants		[23, 50]
	*Lippia citriodora*	Verbena Olorosa (Colombia), Lemon Verbena	Whole plants		[23]
Zingiberaceae	*Boesenbergia rotunda*	Finger Root, Chinese Ginger	Rhizoms	4-hydroxypanduratin A (**1**), panduratin A (**2**)	[58]
Zosteraceae	*Zostera marina*	Marine eelgrass	Whole plants	Zosteric acid (**10**)	[47]

^a^Plants species as yet uninvestigated for anti-dengue activity

## Overview of studies on plant species used as anti-dengue

The use of herbal-based medicine and medicinal plants to treat many diseases is growing worldwide as they has few or no adverse effects. The following sections describe some species of medicinal plants from various families that have been investigated for anti-dengue activity (Table 1). In addition, we describe species used as traditional treatment for dengue together with their isolated compound.

### *Alternanthera philoxeroides*

*Alternanthera philoxeroides* belongs to family Amaranthaceae. *A. philoxeroides* is also called “Alligator Weed”, and is an immersed aquatic plant. It originated from South America but is currently invading Australia. 

The effect of *A. philoxeroides* extracts against dengue virus was investigated in vitro [57]. An MTT assay was carried out to determine the cytotoxicity of *A. philoxeroides* on C6/36 cell lines. Coumarin extract of *A. philoxeroides* showed lowest toxicity on cells (TD_50_ = 535.91), whereas a petroleum ether extract of *A. philoxeroides* had the strongest inhibitory effect on dengue virus (ED_50_ = 47.43).

### *Andrographis paniculata*

*Andrographis paniculata* belongs to family Acanthaceae. It is an erect annual herb native to India and Sri Lanka and cultivated widely in Southern and Southeastern Asia. In Malaysia, it is called “Hempedu Bumi”, which has a bitter taste.

The maximum nontoxic dose (MNTD) of methanolic extract of *A. paniculata* against Vero E6 cells in vitro was investigated [7]. *A. paniculata* recorded the maximal dose, which was not toxic to cells at 0.050^−1^. The methanolic extract of *A. paniculata* showed the highest antiviral inhibitory effect on DENV-1 by antiviral assay based on cytopathic effects.

### *Azidarachta indica*

*Azidarachta indica* belongs to the family Meliaceae. It is fast-growing tree with a final height in the range of 15–20 m. It is native to India and Pakistan and grows throughout tropical and semi-tropical regions.

The in vitro and in vivo inhibitory potential of aqueous extract of *Azidarachta indica* (neem) leaves on the replication of DENV-2 was evaluated [8]. Cytotoxicity studies were carried out to determine the MNTD in a virus inhibition assay. The aqueous extract of neem leaves (NL) completely inhibited 100–10,000 tissue culture infective dose (TCID)_50_ of virus as indicated by the absence of cytopathic effects at its maximum non-toxic concentration of 1.897 mg mL^−1^. An in vivo study on the inhibitory effects on virus of NL aqueous extract in day-old suckling mice was carried out by intracerebral inoculation. It was shown that the aqueous extract inhibited the virus at non-toxic doses in the range of 120–30 mg mL^−1^ as indicated by the absence of 511-bp dengue group specific amplicons upon RT-PCR.

### *Boesenbergia rotunda*

*Boesenbergia rotunda* belongs to family Zingiberaceae. It is a medicinal and culinary herb known as Chinese ginger. It is found throughout China and Southeast Asia.

The activity of some compounds extracted from *B. rotunda* for the inhibition of dengue virus protease has been tested on DENV-2 [58]. The cyclohexenyl chalcone derivatives of *B. rotunda*, 4-hydroxypanduratin A (**1**) and panduratin A (**2**) showed good competitive inhibitory activities towards DENV-2 NS3 protease with *K*_i_ values of 21 μM and 25 μM, respectively. The small value of *K*_i_ shows the potential of 4-hydroxypanduratin A to inhibit DENV-2 NS3 protease in vitro.

### *Carica papaya*

*Carica papaya* belongs to family Caricaceae. It is an erect, fast-growing and unbranched tree or shrub indigenous to Central America and cultivated in Mexico and most tropical countries for its edible fruits.

*C. papaya* leaf has been used traditionally in the treatment of DF [55]. The leaf has been investigated for its potential against DF. The aqueous extract of leaves of this plant exhibited potential activity against DF by increasing the platelet (PLT) count, white blood cells (WBC) and neutrophils (NEUT) in blood samples of a 45-year-old patient bitten by carrier mosquitoes [9]. After 5 days of oral administration of 25 mL aqueous extract of *C. papaya* leaves to the patient twice daily, the PLT count increased from 55 × 10^3^/μL to 168 × 10^3^/μL, WBC from 3.7 × 10^3^/μL to 7.710^3^/μL and NEUT from 46.0 to 78.3 %. Increased platelets could lead to reduced bleeding, thus avoiding progression to the severe illness of DHF.

### *Cladogynos orientalis*

*Cladogynos orientalis* belongs to family Euphorbiaceae. It is a white-stellate-hairy shrub about 2 m high found in Southeast Asia, Malaysia and Thailand.

The in vitro activity of *Cladogynos orientalis*—a Thai medicinal plant—against dengue virus was evaluated [2]. The dichloromethane ethanol extract of *C. orientalis* was tested for anti-dengue activities against DENV-2 in Vero cells by the MTT method. The results showed that the ethanol extract of *C. orientalis* at a concentration of 12.5 μg mL^−1^ exhibited inhibitory activity on DENV-2 with 34.85 %. In addition, *C. orientalis* at a concentration of 100 μg mL^−1^ exhibited an inactivated viral particle activity of 2.9 %.

### *Cladosiphon okamuranus*

*Cladosiphon okamuranus* belongs to family Chordariaceae. It is a brown seaweed found naturally in Okinawa, Japan.

A sulfated polysaccharide named fucoidan (**3**) from *Cladosiphon okamuranus* was found to potentially inhibit DENV-2 infection [59]. The virus infection was tested in BHK-21 cells in a focus-forming assay. Fucoidan reduced infectivity by 20 % at 10 μg mL^−1^ as compared with untreated cells. However, a carboxy-reduced fucoidan in which glucuronic acid was converted to glucose attenuated the inhibitory activity on DENV2 infection.

### *Cryptonemia crenulata*

*Cryptonemia crenulata* belongs to family Halymeniaceae. It is a marine species found throughout the Atlantic Islands, North America, Caribbean Islands, Western Atlantic, South America, Africa, Indian Ocean Islands, Southeast Asia and Pacific Islands.

The sulfated polysaccharides from *Cryptonemia crenulata*, i.e., galactan (**4**), were selective inhibitors of DENV-2 multiplication in Vero cells with IC_50_ values of 1.0 μg mL^−1^, where the IC_50_ values for the reference polysaccharides heparin and DS8000 were 1.9 and 0.9 μg mL^−1^, respectively [60]. However, the compound has lower antiviral effect against DENV-3 and DENV-4, and was totally inactive against DENV-1. The inhibitory effect of C2S-3 against DENV-2 was slightly higher when treatment was by adsorption (EC_50_ = 2.5 ± 0.1 μg mL^−1^) with respect to treatment only during internalization (EC_50_ = 5.5 ± 0.7 μg mL^−1^) [1]. Thus, the inhibitory effect was increased when C2S-3 was included at both stages of adsorption and internalization.

### *Cymbopogon citratus*

*Cymbopogon citratus* belongs to family Poaceae. It is a grass species known as lemon grass and is a tropical plant from Southeast Asia.

The antiviral activity of *Cymbopogon citratus* was determined based on cytopathic effects shown by the degree of inhibition of DENV-1 infected Vero E6 cells [7]. The methanolic extract of *C. citratus* showed a slight inhibition effect on DENV-1. This result was further confirmed with an inhibition assay by the MTT method. However, *C. citrates* showed no significant inhibition. Moreover, *C. citratus* showed the lowest of MNTD at concentration of 0.001 mg mL^−1^. *C. citratus* was found to be quite a cytotoxic plant as it showed maximum cytotoxicity at 0.075 mg mL^−1^.

### *Euphorbia hirta*

*Euphorbia hirta* belongs to family Euphorbiaceae. It is a common weed in garden beds, garden paths and wastelands and is found throughout Java, Sunda, Sumatra, Peninsular Malaysia, the Philippines and Vietnam.

The water decoction of leaves from *Euphorbia hirta*, locally known as gatas–gatas, is used in the Philippines as a folk medicine to treat DF [54]. Internal haemorrhaging will stop and dengue fever will be cured after 24 h. However, the mechanism of action is still unknown and the antiviral properties and its ability to increase blood platelets are currently investigated. The tea obtained from boiled leaves of *E. hirta* is used to cure DF [53].

### *Flagellaria indica*

*Flagellaria indica* belongs to family Flagellariaceae. It is robust perennial climber that grows in many of the tropical and subtropical regions of the Old World, India, Southeast Asia, Polynesia and Australia.

*Flagellaria indica* was investigated for its anti-dengue properties in Vero cells [2]. The antiviral assay results show that 45.52 % inhibition of DENV-2 was observed in vitro in the presence of 12.5 μg mL^−1^ of ethanol extract of the plant. By conducting MTT assays, the cytotoxicity of *F. indica* was determined. The CC_50_ of ethanol extract of *F. indica* were 312 μg mL^−1^. Thus, this study indicates that *F. indica* has a significant potential effect on DENV.

### *Gymnogongrus griffithsiae*

*Gymnogongrus griffithsiae* belongs to family Phyllophoraceae. It is a red seaweed found in Ireland, Europe, Atlantic Islands, North America, South America, Caribbean Islands, Africa, Southwest and Southeast Asia and Australia and New Zealand.

The inhibitory properties against DENV-2 of the sulfated polysaccharide from *Gymnogongrus griffithsiae*, kappa carrageenan (**5**) was evaluated in Vero cells [60]. The compound effectively inhibits DENV-2 multiplication at the IC_50_ value of 0.9 μg mL^−1^, which is the same as the IC_50_ value for the commercial polysaccharides DS8000. However, the compound has lower antiviral effect against DENV-3 and DENV-4, and was totally inactive against DENV-1.

### *Gymnogongrus torulosus*

*Gymnogongrus torulosus* belongs to family Phyllophoraceae. It is a red seaweed found in Australia and New Zealand.

*Gymnogongrus torulosus* was investigated for its in vitro antiviral properties against DENV-2 in Vero cells [61]. Galactan (**4**) extracted from this plant was active against DENV-2, with IC_50_ values in the range of 0.19–1.7 μg mL^−1^.

### *Hippophae rhamnoides*

*Hippophae rhamnoides* belongs to family Elaeagnaceae. It is a deciduous shrub occurring throughout Europe including Britain, from Norway south and east to Spain, and in Asia to Japan and the Himalayas.

The anti-dengue activity of extracts of *Hippophae rhamnoides* leaves was investigated against dengue virus type-2 (DENV-2) in infected blood-derived human macrophages [11]. The findings showed that cells treated with *H. rhamnoides* leaf extracts was able to maintain cell viability of dengue-infected cells on par with Ribavirin, a commercial anti-viral drug along with a decrease and increase in TNF-α and IFN-γ, respectively. Moreover, *H. rhamnoides* leaf extract proved its anti-dengue activity as indicated by a decrease in plaque numbers after the treatment of infected cells.

### *Houttuynia cordata*

*Houttuynia cordata* belongs to family Saururaceae. It is herbaceous perennial flowering plants growing between 20 and 80 cm, and is native to Japan, Korea, Southern China and Southeast Asia.

Ethanol extract from *Houttuynia cordata* revealed an anti-dengue activity with 35.99 % inhibition against DENV-2 in Vero cells at a concentration of 1.56 μg mL^−1^ [2]. Aqueous extract of *H. cordata* showed effective inhibitory action against DENV-2 through direct inactivation of viral particles before infection of the cells [5]. A concentration of 100 μg mL^−1^ also effectively protects the cells from viral entry and inhibits virus activities after adsorption. HPLC analysis of *H. cordata* extract indicated that hyperoside (**6**) was the predominant bioactive compound, and was likely to play a role in this inhibition.

### *Leucaena leucocephala*

*Leucaena leucocephala* belongs to family Fabaceae. It is a species of Mimosoid tree indigenous throughout Southern Mexico and Northern Central America and the West Indies from the Bahamas and Cuba to Trinidad and Tobago.

Galactomannans (**7**) extracted from seeds of *Leucaena leucocephala* have demonstrated activity against yellow fever virus (YFV) and DENV-1 in vitro and in vivo [12]. Galactomannans are polysaccharides consisting of a mannose backbone with galactose side groups, more specifically their structure consists of a main chain of (1 → 4)-linked β-d-mannopyranosyl units substituted by α-d-galactopyranosyl units [62]. *L. leucocephala* show protection against death in 96.5 % of YFV-infected mice. In vitro experiments with DENV-1 in C6/36 cell culture assays showed that the concentration producing a 100-fold decrease in virus titer of DENV-1 was 37 mg L^−1^.

### *Lippia alba* and *Lippia citriodora*

*Lippia alba* and *Lippia citriodora* belong to family Verbenaceae. They are flowering plants native to Southern Texas, Mexico, the Caribbean, Central and South America.

Essential oils from *Lippia alba* and *Lippia citriodora* showed a considerable inhibitory effect on dengue virus serotype replication in Vero cells [23]. A 50 % reduction in virus plaque number values was found with *L. alba* oil at between 0.4–32.6 μg mL^−1^ whereas for *L. citriodora* oil, the IC_50_ values were between 1.9 and 33.7 μg mL^−1^. *L. alba* essential oil was more effective against DENV-2 than other serotypes, while for *L. citriodora* essential oil, the virucidal action against DENV-1, 2 and 3 were similar but lower than against DENV-4. Essential oil of *L. alba* was observed to produce a 100 % reduction of YFV yield at 100 μg mL^−1^ [50].

### *Meristiella gelidium*

*Meristiella gelidium* belongs to family Solieriaceae. It is a marine species found in Atlantic Islands, North America, Caribbean Islands and South America.

The antiviral activity of kappa carragenan (**5**) in *Meristiella gelidium* was evaluated against DENV-2 [63]. The IC_50_ of carragenans isolated from *M. gelidium* was in the range of 0.14–1.6 μg mL^−1^. The results show that the extract and the fraction derived from *M. gelidium* were more effective inhibitors of DENV-2 when compared with reference polysaccharides (heparin and DS 8000).

### *Mimosa scabrella*

*Mimosa scabrella* belongs to family Fabaceae. It is a fast-growing, 15–20 m high and up to 50 cm diameter tree native to the cool, subtropical plateaus of Southeastern Brazil.

Galactomannans (**7**) extracted from seeds of *Mimosa scabrella* have demonstrated activity against YFV and DENV-1 in vitro and in vivo [12]. *M. scabrella* showed protection against death in 87.7 % of YFV-infected mice. In vitro experiments with DENV-1 in C6/36 cell culture assays showed that a concentration of 347 mg L^−1^ produced a 100-fold decrease in virus titer of DENV-1.

### *Momordica charantia*

*Momordica charantia* belongs to family Cucurbitaceae. It is also known as bitter melon or peria (Malaysia), a tropical and subtropical vine found throughout Asia, Africa and the Caribbean.

The MNTD of the methanolic extract of *Momordica charantia* against Vero E6 cells was investigated in vitro [7]. *M. charantia* recorded a maximal dose that was not toxic to cells of 0.20 mg mL^−1^. The methanolic extract of *M. charantia* showed inhibitory effect on DENV-1 by antiviral assay based on cytopathic effects.

### *Ocimum sanctum*

*Ocimum sanctum* belongs to family Labiatae. It is an aromatic herb and shrub native to the tropical regions of Asia and the Americas.

Tea, which is traditionally prepared by using *Ocimum sanctum* boiled leaves, acts as a preventive medicament against DF [64]. The MNTD of methanolic extract of *O. sanctum* against Vero E6 cells in vitro was investigated [7]. However, no significant difference in MNTD for *O. sanctum* was recorded. The methanolic extract of *O. sanctum* showed a slight inhibitory effect on DENV-1 based on cytopathic effects.

### *Piper retrofractum*

*Piper retrofractum* belongs to family Piperaceae. It is a flowering vine native to Southeast Asia and cultivated in Indonesia and Thailand mostly for its fruit.

In vitro anti-dengue activity of *Piper retrofractum* in Vero cells was investigated [2]. The inhibitory activity against DENV-2 infected cells was determined on dichloromethane ethanol extract by the MTT method. The ethanol extract of *P. retrofractum* exhibited an inactivated viral particle activity or 84.93 % at a concentration of 100 μg mL^−1^. Previous study has shown that an aqueous extract of long pepper, *P. retrofractum*, gives the highest level of activity against mosquito larvae [65].

### *Psidium guajava*

*Psidium guajava* belongs to family Myrtaceae. It is an evergreen shrub or small tree indigenous to Mexico, the Caribbean and Central and South America. It is cultivated widely in tropical and subtropical regions around the world.

*Psidium guajava* leaf extract has been tested in vitro and showed to inhibit the growth of dengue virus [66]. Water boiled with guava leaves was used to avoid bleeding in DHF, and increased platelet counts to 100.000/mm^3^ within a period of approximately 16 h [56]. *P. guajava* ripe fruit or juice has healing properties in cases of DF by improving the declining levels of platelets [55].

### *Quercus lusitanica*

*Quercus lusitanica* belongs to family Fagaceae. It is a species of oak native to Morocco, Portugal and Spain.

*Quercus lusitanica* extract was found to have a good inhibitory effect on the replication of DENV-2 in C6/36 cells [39]. The methanol extract of the seeds completely inhibited (10–1,000 fold) the TCID_50_ of virus at its maximum non-toxic concentration of 0.25 mg mL^−1^ as indicated by the absence of cytopathic effects. A low dose of *Q. lusitanica* (0.032 mg mL^−1^) showed 100 % inhibition with 10 TCID_50_ of virus. Proteomics techniqueswere used to demonstrate that the effect of *Q. lusitanica* was to downregulate NS1 protein expression in infected c6/36 cells after treatment with the extract.

### *Rhizophora apiculata*

*Rhizophora apiculata* belongs to family Rhizophoraceae. It is a mangrove tree up to 20 m tall that grows in Australia (Queensland and Northern Territory), Guam, India, Indonesia, Malaysia, Micronesia, New Caledonia, Papua New Guinea, the Philippines, Singapore, the Solomon Islands, Sri Lanka, Taiwan, Maldives, Thailand and Vietnam.

Anti-dengue properties of the ethanolic extract of *Rhizophora apiculata* in DENV-2 in Vero cells have been reported [2]. *R. apiculata* exhibited inhibitory activity and an inactivated viral particle activity of 56.14 % and 41.5 % at concentrations of 12.5 and 100 μg mL^−1^, respectively.

### *Tephrosia crassifolia*, *Tephrosia madrensis* and *Tephrosia viridiflora*

*Tephrosia crassifolia*, *Tephrosia madrensis* and *Tephrosia viridiflora* belong to family Fabaceae. Genus *Tephrosia* is an herb, undershrub or shrub, distributed mainly in tropical and subtropical regions of the world.

Three species from this family (*Tephrosia crassifolia*, *Tephrosia madrensis* and *Tephrosia viridiflora*) were investigated [10]. The flavonoids isolated from *T. madrensis*, glabranine (**8**) and 7-*O*-methyl-glabranine (**9**) exert strong inhibitory effects on dengue virus replication in LLC-MK2 cells. Methyl-hildgardtol A isolated from *T. crassifolia* exhibited a moderate to low inhibitory effect, while hildgargtol A from *T. crassifolia* and elongatine from *T. viridiflora* had no effect on viral growth.

### *Uncaria tomentosa*

*Uncaria tomentosa* belongs to family Rubiaceae. It is a woody vine growing in the tropical jungles of Central and South America.

*Uncaria tomentosa* is a large wood vine native to the Amazon and Central American rainforests [67]. It is used widely as traditional medicine by native people of the Peruvian rainforest [68]. The antiviral activity of *U. tomentosa* was revealed by viral antigen (DENV-Ag) detection in monocytes by flow cytometry in C6/36 cells [67]. The most effective activity emerged from the alkaloidal fraction of *U. tomentosa.* The pentacyclic oxindole alkaloid-enriched fraction of *U. tomentosa* was observed as most effective at decreasing DENV-Ag detection in monocytes at concentrations of 1 μg mL^−1^, whereas the crude hydro-ethanolic extract demonstrates inhibitory activity at concentrations of 10 μg mL^−1^.

### *Zostera marina*

*Zostera marina* belongs to family Zosteraceae. It is an aquatic plant known as eelgrass and is native to North America and Eurasia.

 A compound from the temperate marine eelgrasss *Zostera marina* has been identified as possessing anti-dengue virus activity in a focus-forming unit assay in LLC-MK2 cells [47]. The anti-adhesive compound p-sulfoxy-cinnamic acid, zosteric acid, ZA (**10**), derived from *Z. marina* showed a modest IC_50_ of approximately 2.3 mM against DENV-2. The other compound with related chemistries, CF 238, showed the most activity, with IC_50_ values of 24, 46, 14 and 47 μM against DENV-1, DENV-2, DENV-3 and DENV-4, respectively.

## Summary of medicinal plants tested for their anti-dengue activity

Plants from which extracts have been prepared and tested to detect inhibition activity against DENV are listed in Table 2. This list consists of 16 plant species (from 12 families) that show high anti-dengue activity with high IC_50_ less than 5 μg mL^−1^ on four serotypes of DENV. The plants shown in Table 2 need to be studied further to identify and isolate potential bioactive compounds.

**Table 2:** Some medicinal plants tested for their anti-dengue activity

Family	Species	Part(s) used	Extracts tested	Stage of validation	References
Amaranthaceae	*Alternanthera philoxeroides*	Whole plants	Petroleum ether, ethyl ether, ethyl acetate and coumarin extract	In vitro	[57]
Chordariaceae	*Cladosiphon okamuranus*	Whole plants	Ethanol extract	In vitro	[59]
Euphorbiaceae	*Cladogynos orientalis*	Whole plants	Ethanol extract	In vitro	[2]
Fabaceae	*Leucaena leucocephala*	Seeds	Aqueous extract	In vivo and in vitro	[12, 62]
	*Tephrosia crassifolia*	Leave and flowers	Flavonoid extract	In vitro	[10]
	*Tephrosia madrensis*	Leaves and flowers	Flavonoid extract	In vitro	[10]
	*Tephrosia viridiflora*	Leave and flowers	Flavonoid extract	In vitro	[10]
Fagaceae	*Quercus lusitanica*	Seeds	Methanol extract	In vitro and proteomics technique	[39]
Halymeniaceae	*Cryptonemia crenulata*	Whole plants	Polysaccharide extract	In vitro	[60]
Phyllophoraceae	*Gymnogongrus griffithsiae*	Whole plants	Polysaccharide extract	In vitro	[60]
Piperaceae	*Piper retrofractum*	Whole plants	Dichloromethane and ethanol extract	In vitro	[2, 65]
Rhizophoraceae	*Rhizophora apiculata*	Whole plants	Ethanol extract	In vitro	[2]
Solieraceae	*Meristiella gelidium*	Whole plants	Polysaccharide extract	In vitro	[63]
Verbenaceae	*Lippia alba*	Whole plants	Essential oils	In vitro	[23, 50]
	*Lippia citriodora*	Whole plants	Essential oils	In vitro	[23]
Zosteraceae	*Zostera marina*	–	–	In vitro	[47]

## Potential of plant bioactive compounds to combat dengue

**Fig. 5:**
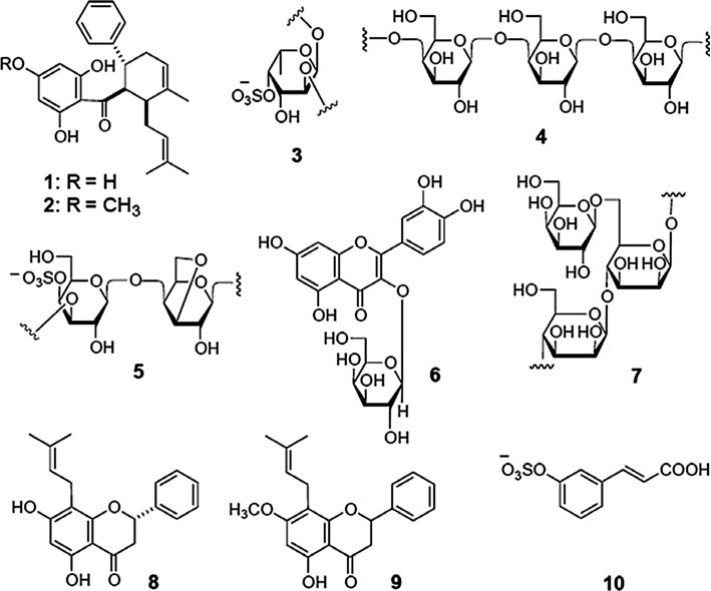
Structure of some potential compounds for treatment of dengue fever (DF) isolated from medicinal plants (**1–10**)

The active compounds showed a wide range of activity against DENV. The isolated products belong to various chemical classes such as sulfated polysaccharides, flavonoids, quercetin and natural chalcone compounds. The chemical structures of ten of these different phytochemicals, isolated from 11 plants, are shown in Fig. 5. The secondary metabolites of medicinal plants comprise a variety of compounds with a wide range of biological activities [68]. There are reports on medicinal plants extracts and essential oils possessing potential to new antiviral properties [41, 42]. Many plant extracts in different solvents have been reported to exhibit activity against a vector of dengue fever, *Ae. Aegypti* [20, 69].

## Conclusion and future directions

This review has covered only 31 potential plants that could be used in the treatment of dengue and about ten isolated active phytochemicals. The available research highlights the information available for various parts and extract types of medicinal plants for the treatment of dengue. However, some of the plants that have not yet been fully explored may have a broad range of potential therapeutic applications. The development of new anti-dengue products from bioactive compounds is necessary in order to find more effective and less toxic anti-dengue drugs. Therefore, any extensive study on the potential of plants with isolated active compounds that have shown anti-dengue activity should go through additional in vitro and in vivo animal testing followed by toxicity and clinical tests. This route may reveal a promising compound to be optimized and thus be suitable for application in the production of new anti-dengue compounds. If pursued from drugs derived from medicinal plants around the continents, this work may prove valuable to the health of individuals and to nations. Moreover, such discoveries may lead to the development of highly efficient and safe anti-dengue treatments. However, to identify potential anti-dengue plants or compounds, knowledge of the mechanisms of virus infection need to be understood inorder to facilitate the search for and development of the most appropriate drugs. Further research is needed to determine how to target the most appropriate stages to prevent the spread of virus infection. Focusing on each phase in the life cycle of the virus, new compounds could prevent (1) infection of host cells, (2) the viral maturation process, (3) synthesis of viral RNA, or (4) the spread of viral particles.

## Abbreviations

CC_50_: Cytotoxicity concentration
DENV: Dengue virus
DF: Dengue fever
DHF: Dengue hemorrhagic fever
DSS: Dengue shock syndrome
HPLC: High performance liquid chromatography
IC_50_: Inhibitory concentration
MNTD: Maximum non-toxic dose
MTT: 3-(4,5-Dimethylthiazol-2-yl)-2,5-diphenyltetrazolium bromide
TCID_50_: Median tissue culture infective dose
